# Cytokine Regulation of Microenvironmental Cells in Myeloproliferative Neoplasms

**DOI:** 10.1155/2015/869242

**Published:** 2015-10-12

**Authors:** Gregor Hoermann, Georg Greiner, Peter Valent

**Affiliations:** ^1^Department of Laboratory Medicine, Division of Hematology & Hemostaseology, Medical University of Vienna, Waehringer Guertel 18-20, 1090 Vienna, Austria; ^2^Department of Internal Medicine I, Division of Hematology & Hemostaseology, Medical University of Vienna, Waehringer Guertel 18-20, 1090 Vienna, Austria; ^3^Ludwig Boltzmann Cluster Oncology, Medical University of Vienna, Waehringer Guertel 18-20, 1090 Vienna, Austria

## Abstract

The term myeloproliferative neoplasms (MPN) refers to a heterogeneous group of diseases including not only polycythemia vera (PV), essential thrombocythemia (ET), and primary myelofibrosis (PMF), but also chronic myeloid leukemia (CML), and systemic mastocytosis (SM). Despite the clinical and biological differences between these diseases, common pathophysiological mechanisms have been identified in MPN. First, aberrant tyrosine kinase signaling due to somatic mutations in certain driver genes is common to these MPN. Second, alterations of the bone marrow microenvironment are found in all MPN types and have been implicated in the pathogenesis of the diseases. Finally, elevated levels of proinflammatory and microenvironment-regulating cytokines are commonly found in all MPN-variants. In this paper, we review the effects of MPN-related oncogenes on cytokine expression and release and describe common as well as distinct pathogenetic mechanisms underlying microenvironmental changes in various MPN. Furthermore, targeting of the microenvironment in MPN is discussed. Such novel therapies may enhance the efficacy and may overcome resistance to established tyrosine kinase inhibitor treatment in these patients. Nevertheless, additional basic studies on the complex interplay of neoplastic and stromal cells are required in order to optimize targeting strategies and to translate these concepts into clinical application.

## 1. Myeloproliferative Neoplasms

Myeloproliferative neoplasms (MPN) are clonal hematopoietic stem cell disorders characterized by abnormal proliferation and expansion of one or more myeloid lineages [1, 2]. The WHO classification of MPN comprises four classic MPN and additional nonclassic MPN. The group of the common, classic MPN includes chronic myeloid leukemia (CML) defined by the Philadelphia chromosome (Ph) and the three Ph-negative entities' polycythemia vera (PV), essential thrombocythemia (ET), and primary myelofibrosis (PMF). The group of nonclassic MPN includes systemic mastocytosis (SM), chronic neutrophilic leukemia (CNL), and chronic eosinophilic leukemia (CEL) [1, 3].

Aberrant tyrosine kinase (TK) signaling is a common hallmark in MPN and has been shown to represent a key driver of the disease. The* BCR-ABL1* fusion gene, which results in a constitutive activation of ABL1 kinase activity, characterizes CML [4–6]. In a majority of patients with PV, ET, and PMF, the activating V617F mutation in the receptor-associated TK* JAK2* is detected [7–10]. In addition, mutations in exon 12 of* JAK2* and mutations in the thrombopoietin receptor (*MPL* W515K/L) have been described in these entities [11, 12]. More recently, somatic mutations in* CALR* were found in* JAK2*- and* MPL*-negative patients with ET or PMF [13, 14]. The activating point mutation D816V in the* KIT* receptor TK is a diagnostic criterion for SM and is found in more than 80% of all patients with SM [15]. A constitutively activated* FIP1L1-PDGFRA* fusion TK has been identified in patients with CEL with or without an accompanying hypereosinophilic syndrome (HES) [16, 17]. More recently,* CSFR3* mutations have been described as a recurrent defect in patients with CNL [18].

Common pathogenic mechanisms are observed despite the variety of different oncogenic mutations underling specific MPN types. Aberrant expression of inflammatory cytokines has been associated with patients' symptoms and alterations of the bone marrow (BM) microenvironment as well as progression of the disease. Several different studies have suggested an important role for the BM microenvironment in the pathogenesis of hematologic malignancies including MPN. In fact, alterations in the BM microenvironment such as increased microvessel density (angiogenesis), fibrosis, and thickening of bone trabeculae are typical pathological findings in MPN and may contribute to disease phenotypes and disease progression. This review focuses on the cytokine regulation of microenvironmental cells with special emphasis on common as well as distinct pathogenetic mechanisms in various MPN. In particular, expression and functional relevance of interleukin-6 (IL-6), IL-8, vascular endothelial growth factor (VEGF), basic fibroblast growth factor (FGF-b), hepatocyte growth factor (HGF), platelet derived growth factor (PDGF), oncostatin M (OSM), tumor necrosis factor-*α* (TNF-*α*), transforming growth factor-*β* (TGF-*β*), and stroma derived factor-1 (SDF-1, CXCL-12) are reviewed. Evidence for increased expression of these cytokines in various MPN is summarized in Table 1. Furthermore, the effect of JAK1/2 inhibitors on the cytokine storm in MPN and targeted drugs for VEGF/VEGFR, HGF/c-MET, and SDF-1/CXCR-4 are discussed.

## 2. Cytokine Expression in Classical MPN

### 2.1. Chronic Myeloid Leukemia

CML is characterized by the reciprocal chromosome translocation t(9;22) and the resulting* BCR-ABL1* fusion gene [5, 6]. The BCR-ABL1 oncoprotein exhibits constitutive TK activity and triggers key signaling pathways, including the RAS-RAF-MEK-ERK pathway, the phosphoinositide 3-kinase-AKT pathway, and STAT5 [19, 20]. Cytokines and other effector molecules downstream of these aberrant signaling cascades have been implicated in the pathogenesis of CML [21].

Aguayo et al. investigated BM vascularity and cytokine levels in CML and other hematologic neoplasms [22]. CML patients reportedly have increased BM vessel density and elevated serum levels of VEGF, HGF, FGF-b, and TNF-*α* compared to controls [23, 24]. Furthermore, high VEGF levels were found to correlate with a shorter survival of patients in chronic phase CML [25]. Immunohistochemical staining of BM sections showed that VEGF is expressed primarily in myeloid progenitor cells, megakaryocytes, and mature granulomonocytic cells in chronic phase CML as well as in myeloid differentiated blast cells in the blast phase of CML [26]. The BCR-ABL1 oncoprotein was found to upregulate expression of VEGF in CML cells, and analysis of signaling pathways downstream of BCR-ABL1 revealed that the mammalian target of rapamycin (mTOR) contributes to BCR-ABL1-dependent expression of VEGF [27]. Targeting of mTOR by rapamycin in CML cells inhibited not only VEGF expression but also the* in vitro* growth of leukemic cells [28]. CD34+ BM cells derived from CML patients secreted up to 10 times more VEGF, FGF-b, HGF, and IL-8 compared to normal donors' BM CD34+ cells. Furthermore, BM mononuclear cells isolated from CML patients induced vascularization of matrigel implants in mice [29]. A number of additional studies described expression of HGF in CML cells [30–33]. In particular, elevated HGF levels in BM and peripheral blood and a correlation of HGF expression with microvessel density in the BM were found. Zhelyazkova and colleagues reported evaluated plasma HGF, cellular HGF, and expression of the HGF receptor c-MET in CML patients. The plasma HGF level correlated with markers reflecting the tumor burden as well as with the phase of CML and overall survival in these patients. In contrast, no prognostic relevance for VEGF levels in chronic phase CML was observed in this study [33]. Also, contrary to VEGF, BCR-ABL1 did not induce synthesis of HGF* in vitro* and targeting of BCR-ABL1 with imatinib showed no effect on HGF expression [34]. Although various cell types may express and release HGF, immunostaining of BM sections revealed that basophils are a primary source of HGF in CML [32]. Expression of PDGF was reported to be associated with BM fibrosis in accelerated and blast phase CML [35].

IL-2 and IL-6 serum levels in patients with CML were found to be significantly elevated compared to controls. Moreover, IL-6 levels in CML patients were found to correlate with BM angiogenesis and reportedly increase during disease progression [51, 52, 92]. The BCR-ABL1 targeting TK inhibitor (TKI) imatinib was found to downregulate IL-6 and IL-8 release in primary CML cells* in vitro* [93]. Hantschel et al. identified IL-8 as one of the strongest downregulated genes in CML upon treatment with the TKI dasatinib [57]. Expression of BCR-ABL1 resulted in a substantial upregulation of IL-8 which was inhibited by dasatinib or nilotinib [57]. TNF-*α* has recently been implicated in stem cell biology of MPN [94, 95]. A study investigated IL-1, IL-6, and TNF-*α* serum levels in CML patients and described no significant difference for TNF-*α* compared to controls [52]. However, CML stem/progenitor cells were found to produce TNF-*α* in a kinase-independent fashion, and at higher levels relative to their normal CD34+ counterparts. In addition, TNF-*α* concentrations were found to be elevated in BM supernatants derived from* BCR-ABL1* transgenic mice compared to wild type mice [95].

### 2.2. Polycythemia Vera, Essential Thrombocythemia, and Primary Myelofibrosis

Elevated levels of inflammatory cytokines have been reported in all entities of classical MPN [38, 49, 51, 53, 58, 96–99]. In particular in PMF, patients suffer from severe constitutional symptoms, and increasing evidence shows that several of these symptoms are mediated by proinflammatory cytokines. Tefferi et al. investigated the prognostic significance of cytokines in PMF by determining serum levels of a comprehensive cytokine panel. In this study, IL-1*β*, IL-1RA, IL-2R, IL-6, IL-8, IL-10, IL-12, IL-13, IL-15, TNF-*α*, granulocyte colony-stimulating factor (G-CSF), interferon *α* (IFN-*α*), macrophage inflammatory protein 1*α* (MIP-1*α*), MIP-1*β*, HGF, IFN-*γ*-inducible protein 10 (IP-10), monokine induced by IFN-*γ* (MIG), monocyte chemotactic protein 1 (MCP-1), and VEGF levels were found to be elevated in PMF patients. In addition, the authors identified IL-8, IL-2R, IL-12, and IL-15 levels as independent prognostic factors for survival of patients with PMF [49]. These findings are in line with other studies showing elevated cytokine level in PMF, ET, and PV [38, 51, 53, 58, 96–98]. However, the methods applied in these studies differed and the panels of elevated cytokines within different studies showed some inconsistences between these studies as reviewed by Hasselbalch [99]. Thus, better standardization is apparently needed to directly compare cytokine production in different MPN cohorts. Nevertheless, increasing evidence indicates that the disease burden of MPN is not only mediated by the primary neoplastic clone but also mediated by a secondary inflammation with an aberrant cytokine production and changes of the BM microenvironment. The concept of cytokines contributing to tissue fibrosis, angiogenesis, and osteosclerosis/osteopenia in MPN has been well established. In particular, FGF-b, IL-8, VEGF, HGF, PDGFR, TGF-*β*, TNF-*α*, and OSM have been implicated in BM microenvironment alterations in patients with MPN [21, 22, 39–41, 68, 69]. Evidence for expression of these cytokines in PV, ET, and PMF is discussed in the next paragraphs.

FGF-b was found to be elevated in the serum of MPN patients. While Musolino et al. reported increased FGF-b levels in PV, ET, and PMF [36], Vaidya et al. found FGF-b—together with IL-1*β*, IL-1RA, IL-2R, EGF, IL-10, FGF-b, IL-12, IFN-*α*, and RANTES—to be particularly elevated in PMF when compared to PV patients [38]. Moreover, high levels of IL-6 and FGF-b were observed in a coculture model of* JAK2* V617F positive hematopoietic cells and stroma cells [34]. Emadi et al. studied IL-8 production in PMF. IL-8 serum levels were significantly increased in patients with PMF, and IL-8 expression was observed in various hematopoietic cell types, including granulocytes, monocytes, megakaryocytic cells, CD34+ progenitor cells, and platelets [100]. Increased serum levels of IL-8 have also been described in patients with PV and ET [38, 58, 101], and IL-8 was found to enhance formation of erythroid colonies* in vitro* [102]. Within a PMF patient cohort, IL-8 serum level was an independent prognostic factor for survival [49].

A number of studies have described elevated VEGF serum levels in MPN [36, 49, 78]. Immunohistochemical studies performed on BM sections of ET, PV, and PMF patients revealed an increased expression of VEGF and its receptor in all MPN groups compared to controls [79, 80]. Megakaryocytes, macrophages, and immature myeloid precursors showed positive immunostaining while erythroid (precursor) cells stained negative for VEGF [80]. Boissinot et al. detected elevated levels of HGF in the serum and BM plasma obtained from PV patients compared to secondary erythrocytosis patients that were employed as controls. Furthermore, BM stem cells and clonal erythroblasts were identified as the major sources of HGF in patients with PV [50]. Further studies analyzing cytokine panels in plasma of MPN patients confirmed elevated HGF levels in PMF, PV, and ET [38, 49].

Wickenhauser et al. described production of TGF-*β* and PDGF in normal human megakaryocytes [103]. Subsequent studies found higher levels of TGF-*β* in megakaryocytes in the BM of patients with myelofibrosis compared to controls. In contrast, no increase in TGF-*β* was found in BM cells of patients with ET [70]. TNF-*α* was found to be elevated in a subset of patients with PMF. Tefferi et al. studied 127 PMF patients and observed significantly higher levels of TNF-*α* compared to controls. However, a substantial number of patients showed no detectable TNF-*α* in peripheral blood and no association with clinical parameters and disease progression was observed [49]. Another study identified TNF-*α* as one of two cytokines that were differentially expressed when stratifying ET and PV patients according to their* JAK2* V617F mutation status [58]. In line with this finding, a murine BM transplant model for* JAK2* V617F showed a marked increase of TNF-*α* serum levels. This increased TNF-*α* level was found to be accompanied by a decrease in erythropoietin and G-CSF, which the authors discussed as a possible suppressive effect of TNF-*α* on normal hematopoiesis [73]. We studied* JAK2* V617F-mediated gene expression and identified IL-6 and the IL-6 family members OSM and leukemia inhibitory factor (LIF) to be directly upregulated by V617F-mutated* JAK2*. Furthermore, oncogene-dependent upregulation of IL-8 and VEGF was observed [59]. Immunohistochemistry staining of BM section from patients with PMF, ET, and PV showed that megakaryocytes, endothelial cells, and myeloid progenitors stain positive for OSM, whereas erythroid cells were OSM negative. This pattern correlates with expression of phosphorylated STAT5, which was identified as the major signaling pathway of oncogene-dependent OSM expression [59].

## 3. Cytokine Expression in Nonclassical MPN: Systemic Mastocytosis

SM is MPN characterized by an abnormal accumulation of mast cells in the BM and other organs [104]. In a substantial subset of patients, SM is accompanied by increased release of various mediators from mast cells and consecutive clinical symptoms [105–107]. The majority of SM patients harbor the somatic* KIT* point mutation D816V. KIT is a receptor TK, and activation of KIT signaling through its ligand stem cell factor (SCF) mediates cell proliferation and survival in immature progenitor cells and mast cell differentiation, as well as mast cells migration, activation, and adhesion [108]. KIT D816V shows constitutively active TK signaling and induces the recruitment of several downstream signaling pathways, including PI3-kinase/AKT [109], mTOR [110], and STAT5 [109, 111].

Brockow et al. measured levels of growth factors in plasma and skin blister fluid of patients with SM [54]. IL-3 and IL-4 were not detectable, and SCF as well as VEGF levels showed no significant difference between patient samples and controls. In contrast, IL-6 was significantly increased in plasma of SM patients and correlated with serum tryptase levels [54]. Subsequent studies confirmed increased IL-6 plasma levels in SM cohorts and suggested a correlation with the severity of symptoms and the presence of osteoporosis [55]. Moreover, IL-6 levels were found to correlate with disease category, severity of BM pathology, organomegaly, and extent of skin involvement. Thus, the authors suggested that IL-6 was a useful surrogate marker of severity of disease [56]. Moreover Rabenhorst et al. investigated cytokines potentially involved in the development of osteopenia or osteoporosis in SM. Again, elevated levels of the proinflammatory cytokine IL-6 were found in patients with SM. High levels of IL-6 were accompanied by increased levels of the osteoclast-regulating factors receptor activator of nuclear factor kappa-B ligand (RANKL) and osteoprotegerin. The authors argue that cytokines produced by mast cells might shift the balance of bone turnover towards increased bone resorption and decreased bone formation [112]. IL-31 has been implicated in the induction of chronic skin inflammation and was found to be increased in patients with SM and to correlate with disease severity [113]. Gene expression studies of purified BM mast cells in SM detected high expression of CCL-23 in indolent and aggressive SM, whereas IL-1*β*, IL-13, or OSM were particularly upregulated in aggressive SM [60].

A number of studies used mast cell lines, in particular the* KIT* D816V mutated human mast cell line HMC-1, to investigate cytokine expression in SM. Selvan and colleagues described expression of MCP-1, MIP-1*α*, MIP-1*β*, RANTES, and IL-8 in HMC-1 cells [114]. Subsequent studies showed expression of TNF-*α* [74], IL-1*β* [74], and OSM [61] in HMC-1 cells. FGF-b was found to be expressed in a number of murine mast cell lines and to be regulated by SCF, TGF-*β*, and TNF-*α* [47]. Immunohistochemical staining of BM sections derived from patients with SM showed expression of FGF-b and in some cases weak expression of TGF-*β* [48]. Furthermore, mast cell infiltrates expressed VEGF as determined by immunohistochemistry of BM sections [88]. Although no significant elevation of VEGF levels was found in plasma of SM patients [54], it is likely that VEGF is locally increased in the BM microenvironment and contributes to increased angiogenesis in SM. Comparative oncology studies in dogs showed expression of VEGF in neoplastic mast cells [89, 90]. Moreover, a correlation of VEGF plasma levels with tumor grade and microvascular density was observed in canine mastocytoma [91].

We studied the effect of* KIT* D816V on cytokine expression in various* in vitro* models. The cytokine profile induced by* KIT* D816V showed a marked overlap when compared to the profile induced by* JAK2* V617F and* FIP1L1-PDGFRA*. A number of cytokines, including OSM, were found to be regulated by all three oncogenes [59, 61, 115]. These studies suggest that the mutant TK in MPN activate common signaling pathways resulting in overlapping effects on cytokine production. Moreover, these and other data indicate that targeting of TK signaling or relevant downstream signaling molecules will reduce the aberrant inflammatory cytokine production not only in PMF, ET, and PV but also in other MPN. A comprehensive analysis of cytokine serum levels in a large cohort of SM patients would be useful to compare the expression of inflammatory cytokines in SM with the pattern observed in other MPN.

## 4. Cytokine Regulation of Microenvironmental Cells

### 4.1. Fibrosis in MPN

Fibrosis is considered to be a reactive process that is often associated with tissue remodeling and tissue repair. Tissue fibrosis may occur in various organs and involves fibroblasts and other connective tissue cells [116]. Concerning development and characteristics of MPN, fibrosis is one of the major pathological findings [117]. The process of fibrosis involves not only local fibroblasts and infiltrating leukocytes resulting in persistence of inflammation in the tissue, but also the proliferation of cells with a myofibroblast phenotype. The pathological mechanisms underlying the development of fibrosis in MPN patients are still not fully understood. Involved cells produce different growth factors, proteolytic enzymes, angiogenic factors, and fibrogenic cytokines, which results in enhancement of connective tissue elements' deposition. This leads to progressively remodeling and finally destruction of physiological tissue architecture [116].

PMF and CML have the highest potential of inducing myelofibrosis. In general, all MPN can develop BM fibrosis, although the likelihood for this varies considerably between the subtypes. The fibrotic potential of MPN with predominant thrombocytosis such as ET can be differentiated from PMF on the basis of morphology. In PMF, the stromal reaction that accompanies clonal hematopoietic stem cell proliferation is characterized by a consistent myelofibrosis associated with osteosclerosis and neoangiogenesis. Thus, fibrosis is a disease-defining hallmark of PMF at diagnosis [118]. In addition, a higher fibrosis grade in patients with PMF correlated with worse prognosis [119]. In patients with PV or ET, reticulin fibrosis at the time of diagnosis is associated with an increased risk of transformation to post-PV or post-ET myelofibrosis [119]. In CML, BM fibrosis occurs in up to 40% of patients at diagnosis and is associated with a poor prognosis [120]. Recently, BM fibrosis in CML was proposed as an independent predictor of responses to TKI therapy [121]. Mastocytosis is also commonly associated with slight-to-moderate BM fibrosis [48, 117]. In the BM of SM patients, mast cell infiltration is often accompanied by fibrosis. In addition, mast cell infiltration with consecutive fibrosis may also occur in the liver, spleen, and lymph nodes [48, 117]. Mast cells produce fibrogenic cytokines including TGF-*β* and FGF-b. Immunohistochemical studies show a close correlation between the mast cell expression of FGF-b and the reticulin fibrosis of mastocytosis lesions [48].

Concerning PMF development, the megakaryocytic lineage seems to play an essential role in promoting myelofibrosis [68]. Megakaryocytic cells were found to produce a variety of growth factors and cytokines leading to proliferation of fibroblast and the development of fibrosis. PDGF is one of the first growth factors that has been implicated in the role of megakaryocytes in development of BM fibrosis [122]. Several studies described increased levels of PDGF in patients with PMF [62, 63], and immunohistochemical staining showed that megakaryocytes and erythroid precursors were highly positive for PDGF [64]. Patients with ET showed increased plasma levels of PDGF; in particular the subgroup of patients with reticulin fibrosis had higher PDGF plasma levels. In contrast, no alteration of intraplatelet PDGF levels was observed in this study [65]. PDGF not only enhances the replication, survival, and migration of myofibroblasts but also modulates the production and secretion of pro- and anti-inflammatory mediators in the pathogenesis of fibrotic diseases [123].

Further studies revealed that the expression and production of TGF-*β* were increased in patients suffering from MPN. Several groups have evaluated TGF-*β* expression in PMF, PV, and ET. These groups reported on quantitative alterations of TGF-*β* and its receptors in megakaryocytic, platelet, and CD34+ progenitor cells and concluded that TGF-*β* was involved in myelofibrosis and myeloproliferation [39, 40, 42, 69–72, 124, 125]. TGF-*β* is a growth factor displaying potent fibrogenic properties and is furthermore associated with not only BM fibrosis, but also clonal hematopoietic expansion and angiogenesis. Moreover, TGF-*β* has been described to negatively regulate progenitor cell growth [126, 127]. In addition, TGF-*β* reportedly promotes the deposition of extracellular matrix in different tissues [128, 129]. In PMF, the pathogenic relevance of TGF-*β* is based on the ability to induce production of types I, III, and IV collagens, fibronectin, tenascin, and proteoglycans. Furthermore, TGF-*β* blocks matrix degradation by reducing collagenase-like protease synthesis, while enhancing protease inhibitor expression [116]. Importantly, TGF-*β* downstream signaling, through SMAD2/3 phosphorylation, has been shown to be active in megakaryocytes extending proplatelets, indicating an autocrine stimulation in megakaryocyte development [125]. TGF-*β* induced PI3-kinase/AKT/NF-*κ*B signaling in hemangioblasts, and activation of this pathway enhanced the production of matrix metalloproteinase-9 [66, 67].

Apart from TGF-*β* and PDGF, FGF-b is considered to be a cytokine with potent fibrogenic characteristics. Several groups analyzed expression of FGF-b in different MPN. The levels of circulating FGF-b were significantly higher in the serum of MPN patients when compared to healthy controls, the highest levels being measured in patients with marked BM fibrosis [37, 39–41, 43, 44, 124]. FGF-b was found to promote fibroblast proliferation in cortical kidney [130]. Furthermore FGF-b promotes cardiac hypertrophy and fibrosis by activating MAPK signaling [131]. Further studies are required to identify the importance of FGF-b in development and progression of MPN. Dalley et al. determined concentration of FGF-b and calmodulin in urine. They showed a significantly elevated calmodulin excretion in PMF patients when compared to PV, ET, and CML. Using a neutralizing antibody to calmodulin influenced the* in vitro* proliferation of normal human fibroblasts. Extracellular calmodulin should also be considered a potential mitogen involved in the stroma cell reaction in patients with PMF [43].

A special situation is* FIP1L1-PDGFRA*+ CEL. In these patients, fibrosis is usually detected in the endomyocardium, which is not the case in other MPN types. One hypothesis is that eosinophils, once entering cardiac tissues, can promote local fibrosis. Eosinophil-related tissue fibrosis has been attributed to infiltration of the tissues with eosinophils and deposition of eosinophil granule proteins [132]. Furthermore, eosinophils were shown to produce the cytokines IL-1*α*, IL-2, IL-3, IL-4, IL-5, IL-6, IL-8, GM-CSF, TGF-*α*, TGF-*β*, TNF-*α*, MIP-1*α*, RANTES, eotaxin, and OSM [133]. Many of the eosinophil-derived cytokines have the potential to stimulate fibroblast proliferation and contribute to local inflammation as well as recruitment of other leukocytes [115, 132]. However, further* in vitro *and* in vivo *models are mandatory to deeply understand the role of cytokines for organ specific fibrosis in CEL.

### 4.2. Angiogenesis in MPN

Angiogenesis, the formation of new vessels from preexisting vessels, plays an important role in development and progression of different tumor types, and targeting of angiogenesis has been successfully translated into clinical practice in various solid tumor models [134]. The process of angiogenesis in hematological malignancies is comparable to the process observed in solid tumors. Endothelial cells from preexisting vessels are activated in the BM by an angiogenic stimulus (e.g., VEGF) and proliferate, migrate, and form new vessels. Initiation of leukemia-induced angiogenesis involves secretion of angiogenic cytokines by leukemic cells and their interaction with the BM stroma [135]. Apart from solid tumors, the importance of angiogenesis becomes increasingly evident in various MPN and other hematologic malignancies. Angiogenesis in the BM of MPN patients was described to correlate with disease burden, progression, and prognosis [36, 79]. Among the classical* BCR-ABL1* negative MPN, increased BM microvessel density (MVD) has been observed in a number of studies in all MPN entities but is most abundant in PMF [45, 75, 80–83, 136]. Not only does PMF show the highest MVD among the classical MPN, but MVD was also described as an independent adverse prognostic factor in PMF [137]. Significantly higher MVD was also found in the BM of patients with post-PV or post-ET myelofibrosis compared to PV or ET [138]. The association of* JAK2* V617F mutation status with MVD showed no significant difference between* JAK2* wild type and mutant MPN patients in two out of three studies [79, 81, 138]. In contrast to the observation that the increase in BM vascularity seems to be generally independent of the* JAK2* V617F status, MVD correlated with* JAK2* V617F mutant allele burden within the* JAK2* V617F+ subgroup [79]. In chronic phase CML, the BM is hypercellular, with a prominent myeloid compartment and left shift in the granulomonocytic cell compartment [23]. Along with myeloid hyperplasia, augmented BM angiogenesis is a typical finding [24, 75]. In particular, the BM of patients with CML shows a significant increase in MVD, functionally associated with elevated levels of angiogenic cytokines [23]. Furthermore, the BCR-ABL1 targeting TKI imatinib was found to reduce the MVD in CML [137]. Alterations of the BM microenvironment are frequently noticed not only in classical MPN but also in SM. These alterations include angiogenesis, thickened bone trabeculae, and sometimes massive BM fibrosis [48, 88, 117, 139]. Our group studied MVD and expression of VEGF in SM. The median BM MVD was found to be significantly higher in SM compared to cutaneous mastocytosis or controls. Furthermore, MVD correlated with the grade of mast cell infiltration in the BM [88].

The process of angiogenesis is tightly controlled by a variety of angiogenic and antiangiogenic cytokines. Leukemic cells upregulate several angiogenic factors leading to increased BM vascularity. VEGF is the most important proangiogenic cytokine that is involved in tumor angiogenesis. VEGF is able to bind to three receptors: VEGF receptor-1 (VEGFR-1; fms-like tyrosine kinase-1, Flt-1), VEGFR-2 (human kinase domain region, KDR/murine fetal liver kinase-1, Flk-1), and VEGFR-3 (Flt-4). VEGFR-2 was found to be both necessary and sufficient to mediate effects of VEGF on endothelial cells, like induction of vascular permeability and angiogenesis [135]. In addition, VEGFR-1 is expressed on hematopoietic stem cells and frequently on leukemic cells [140], whereas megakaryocytes express VEGFR-2 [135], and VEGFR-3 is mainly involved in the regulation of lymphangiogenesis. VEGF not only promotes BM neovascularization but was also found to signal through VEGFRs expressed on the surface of neoplastic hematopoietic cells [141]. Thus, secreted VEGF has been considered to contribute to disease progression by an autocrine or paracrine mechanism [135]. Numerous studies reported increased levels of VEGF in the blood as well as expression in the BM of patients with PV, ET, and PMF [21, 36, 46, 49, 75, 76, 80, 83–87]. Increased expression of VEGF was also found in CML [23, 25, 26, 77] and in BM section of SM patients [88].

HGF and FGF-b are other cytokines with potent angiogenic potential. Endothelial cells express the HGF receptor c-MET and the role of HGF in angiogenesis is well established [142]. HGF enhances vascular matrix degradation and endothelial cell invasion and migration, as well as proliferation of vascular endothelial cells. Furthermore, HGF induces capillary tube formation in a matrigel assay and promotes angiogenesis* in vivo*. HGF acts synergistically with VEGF on endothelial growth but has also been shown to induce angiogenesis independent of VEGF [142]. Elevated levels of HGF have been described in patients with PV, ET, and PMF [38, 49, 50], as well as in CML [30–33]. FGF-b regulates proliferation and function of various mesenchymal cells. It induces growth of fibroblasts and endothelial cells* in vitro* [143] and stimulates angiogenesis and fibrosis* in vivo* [144]. Elevated levels of FGF-b have been described in PV and ET, but particular high levels were found in patients with PMF [36, 38]. In addition, CML patients showed also increased expression of FGF-b [24, 29].

IL-6 is a proinflammatory cytokine that has been implicated in the pathogenesis of various MPN. Among many other functions, IL-6 has been reported to stimulate angiogenesis in the tumor microenvironment and to enhance proliferation and migration of endothelial cells [145–147]. A recent study reported defective pericyte coverage of vessels after IL-6 stimulation compared to VEGF-stimulated vessels [148]. We identified the IL-6 family member OSM as an oncoprotein-dependent cytokine in neoplastic cells of* JAK2* V617F,* KIT* D816V, and* FIP1L1-PDGFRA* positive MPN [59, 61, 115]. OSM has been described to act as a growth factor for various mesenchymal cells, including fibroblasts, osteoblasts, and endothelial cells and to induce angiogenesis* in vitro* and* in vivo* [149–152]. Thus, OSM has been implicated in tissue remodeling, inflammation, and tissue fibrosis [151, 153–155]. Similarly, IL-8 is a multifunctional proinflammatory cytokine which is highly expressed in various MPN. It has been implicated in tumor growth and angiogenesis. In particular, IL-8 was shown to promote endothelial cell proliferation, capillary tube organization, and matrix metalloproteinase expression in endothelial cells [156]. In summary, a number of inflammatory cytokines, abundantly expressed in various MPN, have the potential to trigger angiogenesis in the BM and other organ systems. This pathogenetic process has therefore been proposed as a potential target in CML and other MPN and is best studied for targeted drugs against VEGF/VEGFR and HGF/c-MET.

### 4.3. Bone Marrow Niche Interactions

**Figure 1:**
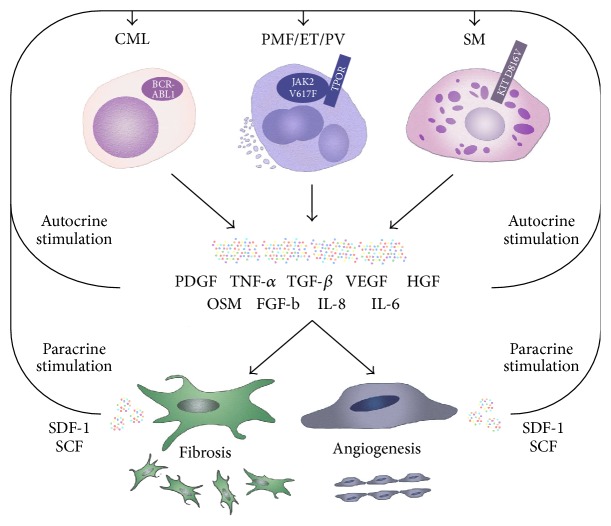
Inflammatory cytokines induce alterations of the bone marrow microenvironment and mediate autocrine and paracrine stimulation of neoplastic cells in myeloproliferative neoplasms. Neoplastic hematopoietic cells in chronic myeloid leukemia (CML), polycythemia vera (PV), essential thrombocythemia (ET), primary myelofibrosis (PMF), and systemic mastocytosis (SM) secrete various cytokines including fibroblast growth factor (FGF), hepatocyte growth factor (HGF), interleukin-6 (IL-6), IL-8, oncostatin M (OSM), platelet derived growth factor (PDGF), transforming growth factor-*β* (TGF-*β*), tumor necrosis factor-*α* (TNF-*α*), and vascular endothelial growth factor (VEGF). These cytokines bind to their receptors on the surface of fibroblast, endothelial cells, and other cells of the bone marrow stroma and induce fibrosis and angiogenesis. In turn, cytokine production in stromal cells (e.g., stroma derived factor-1, SDF-1, or stem cell factor, SCF) has been implicated in proliferation, migration, and clonal selection of hematopoietic cells as well as in resistance to therapy.

Apart from direct effects on endothelial cells and fibroblast, neoplastic cell-derived inflammatory cytokines are also involved in autocrine and paracrine loops between neoplastic (stem) cells and mesenchymal (stem) cells (Figure 1). Hematopoietic stem cells (HSC) rely on their interactions with the BM niche to maintain their quiescent state and to protect their integrity and functions but also to undergo asymmetrical cell division and differentiation in order to regulate and support blood cell production on demand. Similarly, disease-initiating leukemic stem cells (LSC) interact with the BM niche. However, the BM niche in hematopoietic malignancies is commonly altered and the leukemia-induced remodeling of the niche may directly contribute to the aberrant function of LSC [157]. A number of these complex interactions have been described as potentially interesting targets in MPN, of which some are exemplified in this section.

Arranz et al. recently described the effect of nestin-positive mesenchymal stromal cells and sympathetic nerve fibers on the regulation of hematopoietic stem cells in* JAK2* V617F positive MPN. Sympathetic nerve fibers, supporting Schwann cells, and nestin-positive mesenchymal stromal cells were found to be reduced in the BM of MPN patients and murine MPN models, a process that may be triggered by IL-1*β* produced by mutated MPN cells. Depletion of nestin-positive cells or their production of stroma derived factor-1 (SDF-1, CXCL12) accelerated MPN progression. This elegant study demonstrates how inflammatory cytokines produced by neoplastic cells alter or even damage the niche-forming mesenchymal stromal cells in MPN. Furthermore, neuroprotective or sympathomimetic drugs were described as potential therapeutic agents to target this interaction [158].

Expanded myeloid CML cells were found to produce the proinflammatory cytokine IL-6 in inducible* BCR-ABL1* transgenic mouse model recapitulation features of human chronic phase CML. IL-6 served as a positive feedback loop to sustain CML development in this model and reprogrammed both normal and leukemic multipotent progenitor cells towards myeloid development at the expense of lymphoid differentiation. Interestingly, knockout of IL-6 signaling was observed to delay CML development. These results suggest that blocking of IL-6 or targeting the IL-6 signal transduction pathway could represent a valuable target in CML. Moreover, the authors suggested that such self-reinforcing loop—involving IL-6, or other secreted proinflammatory factors—might be relevant in a broad spectrum of MPN [92]. Traer and colleagues studied the effect of the BM microenvironment on imatinib resistance in CML. FGF released from stromal cells was found to promote growth of CML cells through the FGF receptor and the MAP-kinase pathway. In line with the* in vitro* data, CML patients resistant to imatinib without kinase domain mutation showed increased expression of FGF in the BM. Resistance could be overcome with ponatinib, a multikinase inhibitor that targets the FGF receptor in addition to BCR-ABL1 [159]. Another study focused on the effect of stromal cells on the resistance to JAK2 inhibitor treatment in* JAK2* V617F+ disease. Cytokines were found to contribute to this protective effects of stromal cells, and neutralizing antibodies against IL-6, FGF, or CXCL-10 restored the apoptosis induced by JAK2 inhibition [34].

We found that OSM secreted by neoplastic cells did not only stimulate growth of fibroblasts, osteoblasts and microvascular endothelial cells but also induced the production of the angiogenic and profibrogenic cytokines HGF and VEGF in human fibroblasts [59]. In addition, marked production of SDF-1 was induced by OSM in these cells. Thus, specific tumor cell-stroma cell interactions may potentiate the cytokine storm observed in MPN, that is, by inducing the production and release of cytokines that modulate growth of stromal cells as well as their activation, with consecutive expression of additional cytokines and cytokine receptors [58]. Schwaller et al. showed that retroviral overexpression of OSM in BM cells was sufficient to induce a lethal MPN with marked BM fibrosis and polyclonal expansion of myeloid cells [160].

Fleischman and colleagues studied the effect of the proinflammatory cytokine TNF-*α* in MPN.* JAK2* V617F induced TNF-*α* expression in cell lines and primary MPN cells. TNF-*α* in turn was found to reduce colony formation in normal hematopoietic cells while* JAK2* V617F+ progenitor cells were resistant to TNF-*α*. Thus, oncogenic* JAK2* generates a TNF-*α* rich environment which facilitates clonal expansion of mutant cells in MPN [94]. Similarly, CML stem and progenitor cells were found to produce higher levels of TNF-*α* than their normal CD34+ counterparts. TNF-*α* promoted survival of CML stem cells in an autocrine manner by the nuclear factor *κ*B/p65 pathway and expression of IL-3. Importantly, TNF-*α* inhibition induced apoptosis of CML cells and acted synergistically with nilotinib [95]. Together, these findings suggest TNF-*α* as new putative therapeutic target in MPN.

## 5. Targeting the Cytokine Storm and the Microenvironment in MPN: A Novel Concept

### 5.1. JAK Inhibitors and Cytokine Production in MPN

Increased cytokine production was described as a hallmark of classical MPN that contributes to symptom burden of the patients and was referred to as cytokine storm. Targeting of this increased overall cytokine production has been successfully implicated in PMF, PV, and ET. In particular, the identification of the* JAK2* V617F mutation led to the development of various JAK2 inhibitors. Ruxolitinib is the first JAK2 inhibitor approved for treatment of PMF. It targets wild type and mutant JAK2 as well as JAK1 and was found to induce marked and durable reductions in splenomegaly and symptoms in patients with PMF [161]. Despite having only limited effects on the* JAK2* V617F allele burden, significant improvements of fatigue, pain, night sweats, and pruritus were observed after ruxolitinib treatment. In addition, a reduction of cytokine serum levels—including IL-6, IL-8, TNF-*α*, VEGF, and FGF-b—was found. Changes in cytokine level correlated with reduction in spleen size and coincided with symptom improvement [161]. Thus, it is tempting to speculate that the cytokine storm observed in PMF significantly contributes to the symptom burden in PMF. Interestingly, these changes were not related to the patients'* JAK2* mutational status [161]. This is in line with the observation of similar activation patterns of downstream signaling pathways in* JAK2* mutant and wild type cases. The majority of* JAK2* wild type patients harbor* CALR* mutations. Initial observations suggest that mutant* CALR* also activates JAK-STAT signaling [13]. Therefore, targeting of JAK1/JAK2 is effective to reduce proinflammatory cytokines in PMF irrespective of the* JAK2*/*MPL*/*CALR* mutation status [162].

Autocrine GM-CSF stimulation was identified as mechanism of imatinib resistance in CML leading to* BCR-ABL1-*independent activation of JAK/STAT signaling. Wang et al. used the JAK2 inhibitor AG490 to target GM-CSF induced activation of JAK/STAT signaling and could thus overcome resistance to imatinib and nilotinib* in vitro* [163]. Furthermore, activated JAK2/STAT5 signaling has been described as a potential target in LSC in CML [164, 165]. BCR-ABL1 was shown to activate JAK2 and subsequently STAT5 [166]. In addition, BCR-ABL1 was also found to activate STAT5 directly and independently of JAK2, and high levels of STAT5 activation contributed to imatinib resistance [167]. Gallipoli et al. showed that the JAK1/2 inhibitor ruxolitinib synergized with nilotinib in inhibiting the proliferation of CD34+ cells in patients with CML [164]. These findings provide a rationale for the application of JAK2 inhibitors to eradicate residual disease in CML. Clinical trials combining these drugs are now warranted to test this concept in patients.

### 5.2. Targeting of the VEGF/VEGFR Axis

**Figure 2:**
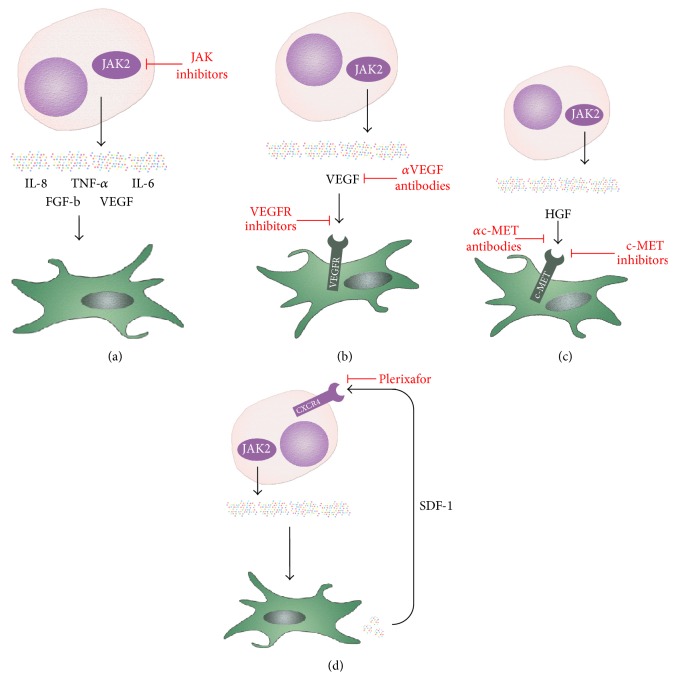
Targeting of cytokines and microenvironment interaction in myeloproliferative neoplasms. (a) JAK inhibitors reduce the expression of various cytokines in MPN. (b) Antibodies against vascular endothelial growth factor (VEGF) and kinase inhibitors targeting the VEGF receptors (VEGFRs) result in disruption of the VEGF/VEGFR axis. (c) Antibodies and kinase inhibitors targeting the hepatocyte growth factor (HGF) receptor c-MET attenuate the HGF/c-MET axis. (d) Plerixafor inhibits the interaction of stroma derived factor-1 (SDF-1) with its receptor CXCR4 which results in mobilization of leukemic stem cells.

Targeting of VEGF and/or the VEGF receptors (VEGFRs) is a widely used concept of antiangiogenesis in oncology. Neutralizing antibodies and soluble receptors are used to inhibit the interaction between VEGF and its receptors (Figure 2(b)). In addition, small molecule inhibitors targeting the kinase activity of VEGFR are applied [168]. Targeting of VEGFR with kinase inhibitors resulted in a reduction in stromal fibroblasts, macrophages, and endothelial cells in* in vitro* cultures of human BM whereas hematopoietic colony formation was not impaired [169].

Bevacizumab is a humanized monoclonal antibody against VEGF approved for antiangiogenic treatment in solid tumors. Mesa and colleagues performed a phase II study enrolling 13 patients with myelofibrosis. None of the patients treated with bevacizumab had an objective response, but significant toxicity was observed. Therefore, this study was terminated early [170]. VEGF promotes angiogenesis mainly through VEGFR-1 and VEGFR-2. Small molecule inhibitors targeting VEGFR and other kinases, for example, sorafenib and sunitinib, have been approved for treatment of patients with renal cell and hepatocellular carcinoma [168]. Sunitinib was tested in a small cohort of patients with PMF. Only one out of 14 patients showed clinical improvement, whereas a high rate of adverse events was observed [171]. Vatalanib is a VEGFR kinase inhibitor with greater potency against VEGFR-2 than against VEGFR-1 or VEGFR-3. In addition, inhibitory effects on PDGF receptor and KIT are observed. A phase I study in PMF showed modest activity with clinical improvement in 20% of the patients examined [172].

mTOR was identified to mediate* BCR-ABL1*-dependent VEGF expression in CML [27]. Targeting of mTOR by rapamycin in CML cells inhibited not only VEGF expression but also the* in vitro* growth of leukemic cells [28]. A clinical pilot study to evaluate the antileukemic and antiangiogenic effects of rapamycin in patients with imatinib-resistant CML showed transient antileukemic effects in a subset of cases [173]. In summary, despite promising data in preclinical models, direct targeting of VEGF resulted only in modest clinical effects on patients with MPN so far.

### 5.3. Targeting of the HGF/c-MET Axis

Aberrant activation of HGF and/or its receptor c-MET has been described in solid tumors as well as in acute myeloid leukemia (AML), myeloma, and MPN. Production of HGF was found to be independent of* BCR-ABL1* in CML and independent of* JAK2* V617F in other MPN [32]. Thus, blocking of the HGF/c-MET function was suggested as an independent therapeutic target which could synergize with TKI treatment in MPN [174].

c-MET neutralizing antibodies bind to the extracellular domain of the receptor and prevent binding of HGF to c-MET (Figure 2(c)) [160]. These antibodies have shown promising effects on solid tumors [174].* In vitro* studies have shown that c-MET neutralizing antibodies can effectively suppress the growth of* JAK2* V617F-mutated cells, including PV erythroblasts and the HEL cell line, which expresses HGF at high levels [50]. In addition, small molecule inhibitors targeting c-MET and the c-MET-related RON receptor have been developed. The c-MET inhibitors SU-11274 and PHA-665752 decreased the survival of AML cells in a dose dependent manner [174]. SU-11274 was found to inhibit colony formation, to reduce viability, and to induce differentiation in A9M, U937, and OCI-AML cells [175]. Moreover, the c-MET inhibitors were found to block the response to HGF in a myeloma model [176]. Our group tested the effects of SU-11274 and PF-02341066 (crizotinib) on* BCR-ABL1* positive cells and found that both drugs induce a significant growth reduction in KU812 cells and K562 cells [34]. Furthermore, c-MET inhibitors were found to reduce proliferation of primary CML cells* in vitro* [32]. Boissinot and colleagues tested the efficacy of combining c-MET and JAK inhibitors on the proliferation of the* JAK2 *V167F positive HEL and UKE-1 cell lines. Only a weak inhibition was observed when molecules were tested separately, whereas the combination of the c-MET inhibitor PF-2341066 and the JAK inhibitor ruxolitinib inhibited growth of UKE-1 cells [174]. In summary, preclinical models show promising results for HGF/c-MET inhibition in MPN. The clinical efficacy of this targeting approach remains to be tested in clinical trials.

### 5.4. Targeting of the SDF-1/CXCR4 Axis

Increasing evidence suggests an important role of the BM microenvironment in the regulation of proliferation and survival of normal and leukemic hematopoietic stem cells. Thus, targeting of the specific BM niches and stem cell-niche interactions has been suggested as a promising therapeutic strategy [177]. SDF-1 (CXCL-12) is a chemokine produced by mesenchymal cells of the BM stroma (e.g., endothelial cells and osteoblasts) with particularly high expression in perivascular, niche-forming mesenchymal stromal cells [178]. Hematopoietic stem and progenitor cells express the SDF-1 receptor CXCR4 and migrate specifically towards SDF-1. Plerixafor (AMD3100) inhibits the SDF-1/CXCR4 interaction and is clinically used to mobilize hematopoietic stem and progenitor cells in stem cell transplant donors [179]. The SDF-1/CXCR4 axis is one potential target in the interplay of leukemic stem cells (LSC) and the BM microenvironment (Figure 2(d)).

CXCR4 is highly expressed on the surface of malignant cell in chronic lymphocytic leukemia (CLL), and SDF-1 was found to promote chemotaxis of CLL cells and their interaction with stromal cells, which was shown to induce resistance of CLL cells to cytotoxic agents, and was furthermore suggested to mediate persistence of minimal residual disease in the BM during therapy. In line with this concept, CXCR4 antagonists were successfully used to block interactions between CLL and stromal cells and to mobilize CLL cells from their protective microenvironments, becoming thus accessible to conventional drugs [180]. Similar targeting concepts were applied in preclinical models for AML and acute lymphoblastic leukemia (ALL). CXCR4 antagonist inhibited the proliferation of AML cells and reduced protection against chemotherapy by stromal cells* in vitro* and* in vivo* [181–183]. Leukemic cells in T-ALL were found to be in direct, stable contact with SDF-1-producing BM stroma. Furthermore, genetic targeting of CXCR4 in murine T-ALL led to rapid, sustained disease remission and CXCR4 antagonism suppressed human T-ALL in primary xenograft models [184].

Partly ambivalent results have been published for the role of SDF-1/CXCR4 in MPN, and although increased levels of SDF-1 have been reported, this may not necessarily result in a sustained activation of CXCR4 signaling in neoplastic cells [185, 186]. On the one hand, mobilization of CD34+ cells in patients with PMF has been attributed to reduced CXCR4 expression and hypermethylation of the CXCR4 promoter [187, 188]. Moreover, although elevated levels of immunoreactive forms of SDF-1 were found in the BM and peripheral blood of patients with PMF and PV, detailed studies using mass spectrometry have shown that SDF-1 was mainly truncated and thus expressed in an inactive form in these patients. The authors of this study concluded that reduced levels of intact SDF-1 due to proteolytic degradation would contribute to the mobilization of hematopoietic stem cells in PMF [186]. In line with these data, CD34+ cells in CML showed an impaired chemotactic response to SDF-1 although no decrease in CXCR4 expression was observed [189, 190]. Our group identified the cell surface enzyme dipeptidylpeptidase-IV (CD26) as a marker of CML LSC. CD26 was shown to disrupt the SDF-1/CXCR4 axis by cleaving SDF-1, and targeting of CD26 by gliptins suppressed the expansion of* BCR-ABL1*+ cells. CD26 expression may explain the mobilization of LSC and the observed extramedullary spread of hematopoietic stem and progenitor cells in CML, and inhibition of CD26 may revert abnormal LSC function [191].

On the other hand, the SDF-1/CXCR axis between stroma and leukemic cells contributes to resistance to TKI treatment in CML. Imatinib was found to enhance migration of CML cells towards stromal cell layers, which may in turn promote nonpharmacological resistance to imatinib [192, 193]. Mechanistically, this finding was linked to CXCR4 redistribution into the lipid raft fraction, in which CXCR4 colocalized with active LYN after TKI treatment [193]. The CXCR4 inhibitor plerixafor diminished migration of* BCR-ABL1* positive cells and reduced adhesion of these cells to extracellular-matrix components and to BM stromal cells* in vitro*. Moreover, plerixafor was also found to decrease the drug resistance of CML cells induced by coculture with BM stromal cells* in vitro*. Importantly, plerixafor was shown to mobilize leukemic cells* in vivo* and to act synergistically with nilotinib to reduce the leukemia burden in a mouse model. The authors of this study argue that the combination of CXCR4 inhibition with TKI treatment in CML might be a useful approach to override drug resistance and to achieve deeper responses in CML [194]. In contrast, another study tested the effects of plerixafor in combination with either imatinib or dasatinib in a murine CML BM transplant model. In this study, no beneficial effect of plerixafor over TKI monotherapy was observed. Moreover, an increase in CNS infiltration after plerixafor treatment was described [195]. The discrepancy of these date can partly be explained by difference in the CML mouse model (e.g., irradiation possibly contributing to CNS infiltration) and in the TKI administration. Weisberg et al. applied plerixafor after marked reduction of disease burden with nilotinib as a model of minimal residual disease and argued that the absence of significant disease burden was relevant for the beneficial effects of the combination therapy [194].

Thus, the SDF-1/CXCR4 axis is a promising but still controversial target in CML and other types of MPN. The effect of CXCR4 inhibitors in PV, ET, PMF, and SM remains to be addressed in further preclinical models.

## 6. Concluding Remarks and Future Perspectives

The complex interplay between neoplastic cells and microenvironmental cells in MPN has gained increasing interest in recent years. The resulting research revealed new important insights into the pathogenesis of MPN. One important aspect is that oncogenic signaling promotes cytokine production in MPN cells and alters their interaction with the BM stroma. A number of pathogenetic mechanisms are found to be conserved between various MPN, and lessons learned from one disease can be exploited for the other MPN types. Thus, it will be important to compare systematically the various common as well as rare MPN-variants in terms of basic and clinical science.

More recently, the pathologically altered interactions between neoplastic cells and their microenvironment have been investigated with the aim of defining new potential targets of therapy and to develop novel therapeutic approaches. First, the increased angiogenesis and BM fibrosis may serve as novel targets of therapy in MPN. Indeed, several TKI used to treat MPN may also suppress angiogenesis and/or fibrosis through inhibition of vascular target kinases. Thus, the VEGF/VEGFR, HGF/c-MET, and SDF-1/CXCR4 axis are potential targets in MPN, and a number of other molecular targets are under investigation. Many open questions still have to be addressed in preclinical model, and so far only few of the many exciting approaches were successfully translated to the clinic. Best evidence for targeting of the inflammatory cytokine storm is derived from the clinical efficacy of JAK inhibitors in MPN, which show marked benefits in patients despite their lack of specificity for mutant JAK2. Other targeting approaches for inflammatory cytokines will most likely be combined with established or experimental inhibitors of the primary oncoprotein in the given MPN. Increasing knowledge of the LSC-niche interaction will help to optimize this combined targeting approach and to establish synergistic strategies for therapy or even cure of MPN.

## Figures and Tables

**Table 1:** Increased expression of cytokines in myeloproliferative neoplasms. Evidence for increased expression of the cytokines fibroblast growth factor (FGF), hepatocyte growth factor (HGF), interleukin-6 (IL-6), IL-8, oncostatin M (OSM), platelet derived growth factor (PDGF), transforming growth factor-*β* (TGF-*β*), tumor necrosis factor-*α* (TNF-*α*), and vascular endothelial growth factor (VEGF) in the myeloproliferative neoplasms chronic myeloid leukemia (CML), polycythemia vera (PV), essential thrombocythemia (ET), primary myelofibrosis (PMF), and systemic mastocytosis (SM) is shown. The numbers indicate selected references for elevated expression of the cytokine in the given myeloproliferative neoplasm.

Disease	CML	PV, ET, PMF	SM
Oncogene	*BCR-ABL1*	*JAK2* V617F, *CALR*, *MPL*	*KIT* D816V
FGF	[31, 36, 37]	[36–46]	[47, 48]
HGF	[30, 31, 33]	[38, 39, 49, 50]	
IL-6	[51, 52]	[38, 49, 51, 53]	[54–56]
IL-8	[57]	[49, 53, 58]	
OSM		[59]	[60, 61]
PDGF	[35]	[22, 46, 62–65]	
TGF-*β*	[66, 67]	[40, 42, 46, 68–72]	[48]
TNF-*α*	[31, 52]	[38, 49, 73]	[74]
VEGF	[26, 27, 29, 31, 33, 36, 75–77]	[21, 36, 38, 45, 46, 49, 58, 75, 76, 78–87]	[54, 88–91]
